# Specimen preparation for cryogenic coherent X-ray diffraction imaging of biological cells and cellular organelles by using the X-ray free-electron laser at SACLA

**DOI:** 10.1107/S1600577516007736

**Published:** 2016-05-31

**Authors:** Amane Kobayashi, Yuki Sekiguchi, Tomotaka Oroguchi, Koji Okajima, Asahi Fukuda, Mao Oide, Masaki Yamamoto, Masayoshi Nakasako

**Affiliations:** a Department of Physics, Faculty of Science and Technology, Keio University, 3-14-1 Hiyoshi, Kohoku-ku, Yokohama, Kanagawa 223-8522, Japan; b RIKEN SPring-8 Center, 1-1-1 Kouto, Sayo, Hyogo 679-5148, Japan

**Keywords:** coherent X-ray diffraction imaging, X-ray free-electron laser, frozen-hydrated non-crystalline specimens, cryogenic X-ray diffraction experiment, structures of cells and cellular organelles

## Abstract

Coherent X-ray diffraction imaging (CXDI) allows internal structures of biological cells and cellular organelles to be analyzed. CXDI experiments have been conducted at 66 K for frozen-hydrated biological specimens at the SPring-8 Angstrom Compact Free-Electron Laser facility (SACLA). In these cryogenic CXDI experiments using X-ray free-electron laser (XFEL) pulses, specimen particles dispersed on thin membranes of specimen disks are transferred into the vacuum chamber of a diffraction apparatus. Because focused single XFEL pulses destroy specimen particles at the atomic level, diffraction patterns are collected through raster scanning the specimen disks to provide fresh specimen particles in the irradiation area. The efficiency of diffraction data collection in cryogenic experiments depends on the quality of the prepared specimens. Here, detailed procedures for preparing frozen-hydrated biological specimens, particularly thin membranes and devices developed in our laboratory, are reported. In addition, the quality of the frozen-hydrated specimens are evaluated by analyzing the characteristics of the collected diffraction patterns. Based on the experimental results, the internal structures of the frozen-hydrated specimens and the future development for efficient diffraction data collection are discussed.

## Abstract 2

Detailed procedures and key points in preparing frozen-hydrated biological specimens are reported for efficient cryogenic coherent X-ray diffraction imaging using an X-ray free-electron laser.

## 1. Introduction   

Coherent X-ray diffraction imaging (CXDI) has been applied to visualize the internal structures of non-crystalline particles with dimensions of micrometers to sub-micrometers in materials science and biology (Miao *et al.*, 1999 ▸, 2015 ▸). In CXDI experiments, a spatially isolated non-crystalline particle is irradiated by coherent X-rays, and then the Fraunhofer diffraction pattern of the particle is recorded by an area detector under the oversampling condition (Miao *et al.*, 2003*a* ▸). From the small-angle diffraction pattern, the electron density map of the particle projected along the direction of incident X-rays is, in principle, restored by applying the iterative phase-retrieval algorithm (Fienup, 1982 ▸). The long penetration depth of short-wavelength X-rays allows us to visualize the internal structures of whole specimen particles, which are opaque in transmission electron microscopy (EM), at resolutions higher than optical microscopy (Miao *et al.*, 2002 ▸, 2003*b* ▸, 2005 ▸; Robinson *et al.*, 2001 ▸; Song *et al.*, 2008 ▸; Nishino *et al.*, 2009 ▸; Jiang *et al.*, 2010 ▸; Takayama & Nakasako, 2012 ▸; Nakasako *et al.*, 2013 ▸; Gallagher-Jones *et al.*, 2014 ▸).

Now, X-ray free-electron lasers (XFELs) are leading CXDI to a new stage. X-ray pulses with complete spatial coherence and duration of tens of femtoseconds are available at XFEL facilities such as the Linac Coherent Light Source (LCLS; Emma *et al.*, 2010 ▸) and the SPring-8 Angstrom Compact Free-Electron Laser (SACLA; Ishikawa *et al.*, 2012 ▸). We can collect diffraction data before radiation-damage processes occur (Chapman *et al.*, 2006*a* ▸; Hirata *et al.*, 2014 ▸), although specimens irradiated by single XFEL pulses are destroyed at the atomic level (Chapman *et al.*, 2006*a* ▸; Nakasako *et al.*, 2013 ▸). Owing to the repetition rate of XFEL pulses of 30–120 Hz, a huge number of diffraction patterns can be collected within a short period of time.

In XFEL-CXDI structural studies, an important target is non-crystalline biological specimens such as cells and cellular organelles. In diffraction experiments, specimens are set in a vacuum to avoid absorption and scattering of the incident and diffracted X-rays by air. However, a vacuum environment causes fatal damage to biological specimens, which require aqueous environments to maintain their functional structures. In the XFEL-CXDI field, the liquid jet method (Kassemeyer, 2014 ▸), the aerosol method (Seibert *et al.*, 2011 ▸; van der Schot *et al.*, 2015 ▸) and the micro-liquid enclosure-array method (Kimura *et al.*, 2014 ▸) have been developed to collect diffraction patterns from biological specimens in vacuum chambers of diffraction apparatus. To date, these experimental methods have been applied to the structure analyses of viruses (Seibert *et al.*, 2011 ▸; Kassemeyer *et al.*, 2012 ▸; Ekeberg *et al.*, 2015 ▸), cellular components (Hantke *et al.*, 2014 ▸) and bacterial cells (Kimura *et al.*, 2014 ▸; van der Schot *et al.*, 2015 ▸).

As an alternative approach to the three methods mentioned above, we proposed XFEL-CXDI experiments for frozen-hydrated biological specimens at cryogenic temperatures (Nakasako *et al.*, 2013 ▸; Oroguchi *et al.*, 2015 ▸; Kobayashi *et al.*, 2016 ▸). It has been demonstrated previously that cryogenic technologies enable us to store biological cells at low temperatures, for instance, in medical science (Chen, 1986 ▸; Medeiros *et al.*, 2002 ▸; Schwartz, 1986 ▸). Frozen-hydrated cells and cellular organelles keep their functional structures (McDowall *et al.*, 1983 ▸), and are still alive after returning to ambient temperatures (Gibson & Khoury, 1986 ▸).

Cryogenic specimen preparation enables us to store frozen-hydrated specimens in liquid nitrogen for CXDI experiments. Therefore, we can harvest a large amount of biological cells and isolated unstable cellular organelles at a desired period of the cell cycle. Moreover, frozen-hydrated specimens are free from adiabatic expansion, bubbling of water inside biological specimens, evaporation and drying even under vacuum conditions. The rate of XFEL pulses hitting single specimen particles can be controlled by the number density of particles dispersed on membranes as described below.

**Figure 1:**
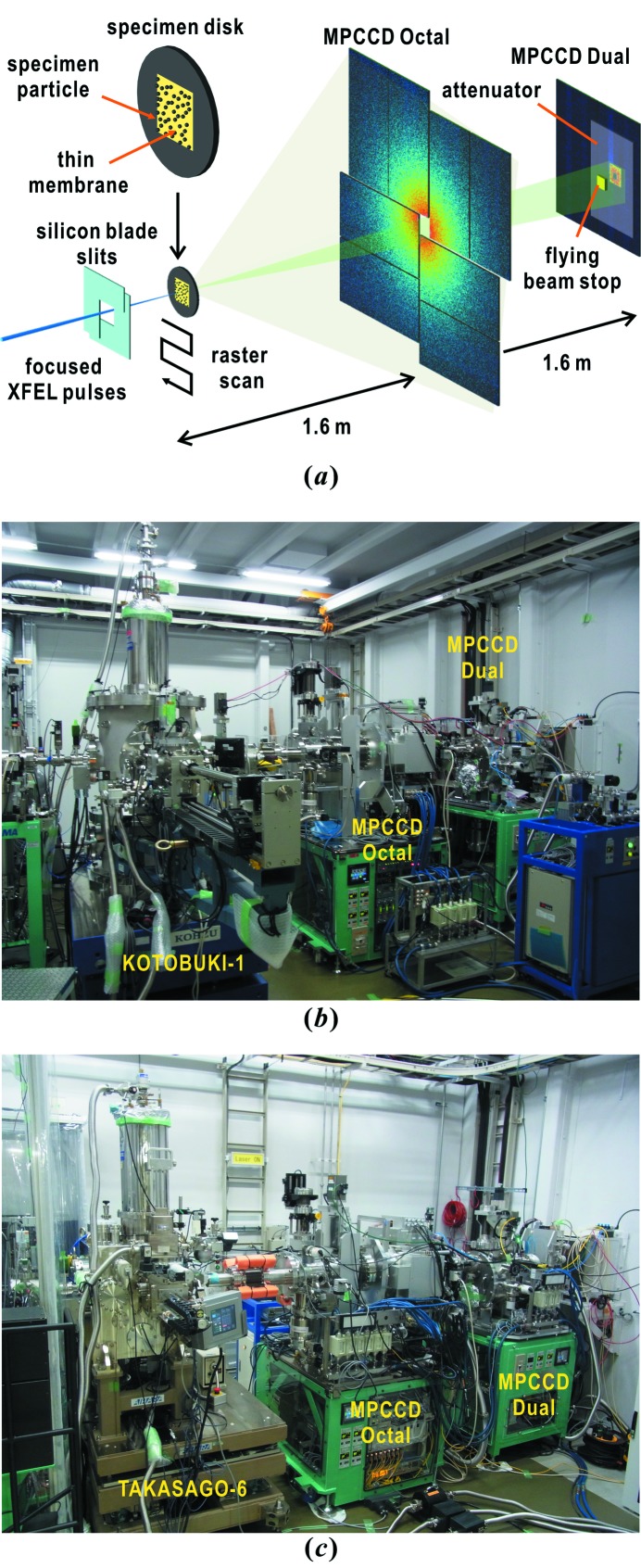
(*a*) Schematic illustration of cryogenic XFEL-CXDI experiments by raster scanning of specimen disks with non-crystalline specimen particles adsorbed onto the thin membrane region. Photographs of the KOTOBUKI-1 diffraction apparatus connected to the two MPCCD detectors in experimental hutch 3 (*b*) and the TAKASAGO-6 with the two MPCCD detectors in hutch 4 (*c*) of BL3 at SACLA.

We have conducted a series of cryogenic XFEL-CXDI experiments for frozen-hydrated non-crystalline biological specimens, such as cells and cellular organelles, using our custom-made diffraction apparatus at SACLA (Fig. 1 ▸) (Nakasako *et al.*, 2013 ▸; Oroguchi *et al.*, 2015 ▸; Takayama *et al.*, 2015 ▸; Kobayashi *et al.*, 2016 ▸). In our XFEL-CXDI experiments, the specimen particles dispersed on thin membranes of specimen disks are flash-cooled into the frozen-hydrated state. The specimen disks are then transferred into a vacuum chamber of the diffraction apparatus using specially designed devices. Focused single XFEL pulses destroy specimen particles at the atomic level and then particles within a diameter of approximately 10 µm from the center of focused XFEL pulses are degraded or expelled from the membrane surfaces (Nakasako *et al.*, 2013 ▸; Kobayashi *et al.*, 2016 ▸). Therefore, diffraction patterns are collected through raster scanning specimen disks in 25 µm steps to provide fresh specimen particles in the irradiation area (Nakasako *et al.*, 2013 ▸; Kobayashi *et al.*, 2016 ▸).

We have developed preparation procedures of frozen-hydrated biological specimens for cryogenic XFEL-CXDI experiments by referring to the sophisticated specimen preparation procedures in cryogenic EM (Robarts & Sleytr, 1985 ▸; Grassucci *et al.*, 2007 ▸) and cryogenic protein X-ray crystallography (Nakasako, 1995 ▸). Here, we report the procedures including the materials and devices used, and evaluate the quality of the prepared specimens through analyzing the diffraction patterns from frozen-hydrated cyanobacterial cells. The methods described here would be useful for imaging the structures of non-crystalline particles in CXDI experiments.

## 2. Methods   

### 2.1. Membranes and specimen disks   

**Figure 2:**
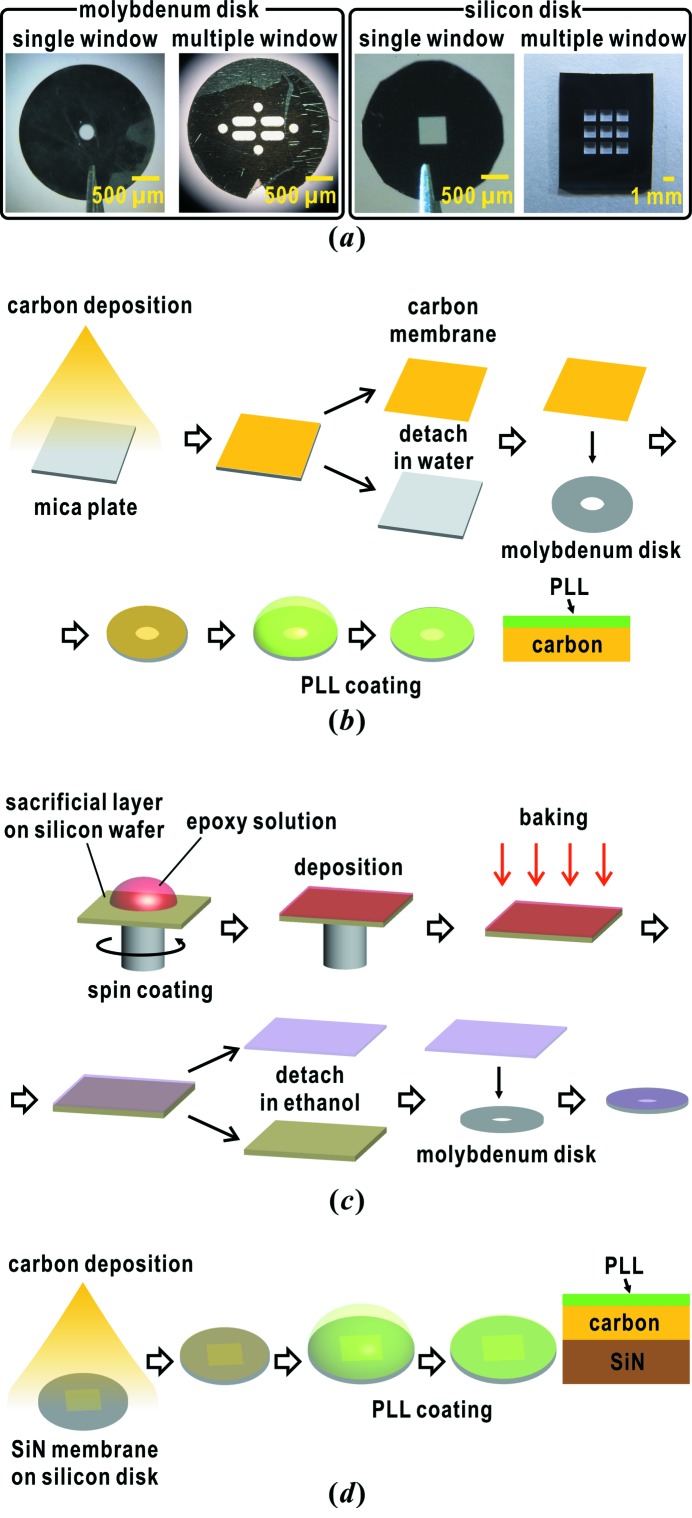
(*a*) Two types of specimen disks used in our XFEL-CXDI experiments. Schematic illustrations on the preparation procedures of specimen disks with carbon membranes (*b*), epoxy membranes (*c*) and SiN membranes (*d*).

In our specimen preparation, specimen particles are dispersed on thin membranes attached to specimen disks under moist conditions. The specimen disks are flash-cooled by liquid ethane at a cooling rate of ∼3700 K s^−1^ (Nakasako, 1995 ▸). Therefore, the membranes and specimen disks are required to be able to withstand flash-cooling. In addition, diffraction intensities from the membranes must be negligibly weak in comparison with those from the specimen particles. To date, we have used molybdenum disks (Okenshoji, Japan) with single or multiple windows of carbon or chemically synthesized membranes (Fig. 2*a* ▸). Because of their small thermal contraction, molybdenum disks are used in cryogenic EM (Grassucci *et al.*, 2007 ▸). We have also used silicon disks with single or multiple silicon nitride (SiN) membrane windows (Norcada, Canada), which are able to withstand flash-cooling by liquid ethane (Fig. 2*a* ▸).

Carbon membranes of approximate thickness 30 nm are created on the surfaces of freshly cleaved mica plates of approximate size 5 mm × 5 mm by using a JEE-420 vacuum evaporator (JEOL, Japan) (Fig. 2*b* ▸). The thickness of the carbon membrane is controlled by monitoring the degree of carbon deposition on white filter paper placed near the mica plates. The carbon membranes are then detached from the mica plates at the air–water interface when inserting the carbon-deposited mica plates into water. The carbon membranes floating on the water are picked up by custom-made molybdenum specimen disks (Fig. 2*a* ▸) such that a single carbon film covers the windows without wrinkles.

Thermoset polymer membranes, hereafter designated as epoxy membranes, are chemically synthesized according to the previously published protocol (Watanabe & Kunitake, 2007 ▸) (Fig. 2*c* ▸). Poly[(*o*-cresyl glycidyl ether)-*co*-formaldehyde] (Sigma-Aldrich, USA) and polyethylenimine (Sigma-Aldrich, USA) are mixed in chloroform. A droplet of the mixture is applied to a sacrificial layer of poly(4-hydroxystyrene) (Sigma-Aldrich, USA) prepared on a silicon wafer. To make a thin layer of the mixture, the wafer is rotated at a speed of 7500 r.p.m. for 1 min using a MS-A100 spin coater (Mikasa, Japan). Then, the wafer is baked at 393 K for 5 min in a BO-R65JB oven (Mitsubishi Electric, Japan). The thickness of the membrane can be controlled by the ratio of the weight of the polymers to the volume of chloroform in the range 0.1–1% (*w*/*v*), and is measured using an SP400 atomic force microscope (Seiko Instruments, Japan) and/or a MiniScope TM3000 scanning electron microscope (Hitachi High-Technologies, Japan). The sacrificial layer is dissolved in ethanol after being trimmed to an approximate size of 5 mm × 5 mm. Then, the epoxy membranes are detached from the silicon wafer. The epoxy membranes, having been transferred into water, are then picked-up by the molybdenum specimen disks (Fig. 2*a* ▸).

In addition to the laboratory-made thin membranes, we use two types of silicon disks which have windows of SiN membrane of thickness 100 nm (Fig. 2*a* ▸). One type is commercially available silicon disks of diameter 3 mm with one 500 µm × 500 µm window (Norcada, Canada). On the other hand, we use custom-made silicon disks of size 10 mm × 8 mm with nine windows of 1 mm × 1 mm (Norcada, Canada) for collecting a huge number of diffraction patterns from the specimen particles.

Carbon membranes and SiN membranes are hydrophobic, while epoxy membranes have high affinity to biological specimens because of their hydrophilicity. Therefore, carbon membranes are treated with a 0.1 mg mL^−1^ solution of poly-l-lysine (PLL) with a molecular weight of approximately 300 kDa (Sigma-Aldrich, USA) to increase their affinity to biological specimens (Fig. 2*b* ▸). After 30 min of treatment, unbound PLL is washed out using distilled water. Because SiN membranes have little affinity with PLL, they are coated with carbon layers of thickness 15 nm prior to the PLL decoration (Fig. 2*d* ▸). The PLL treatment dramatically increases the affinity of these membranes to cells and organelles.

### 2.2. Preparation of standard specimens   

**Figure 3:**
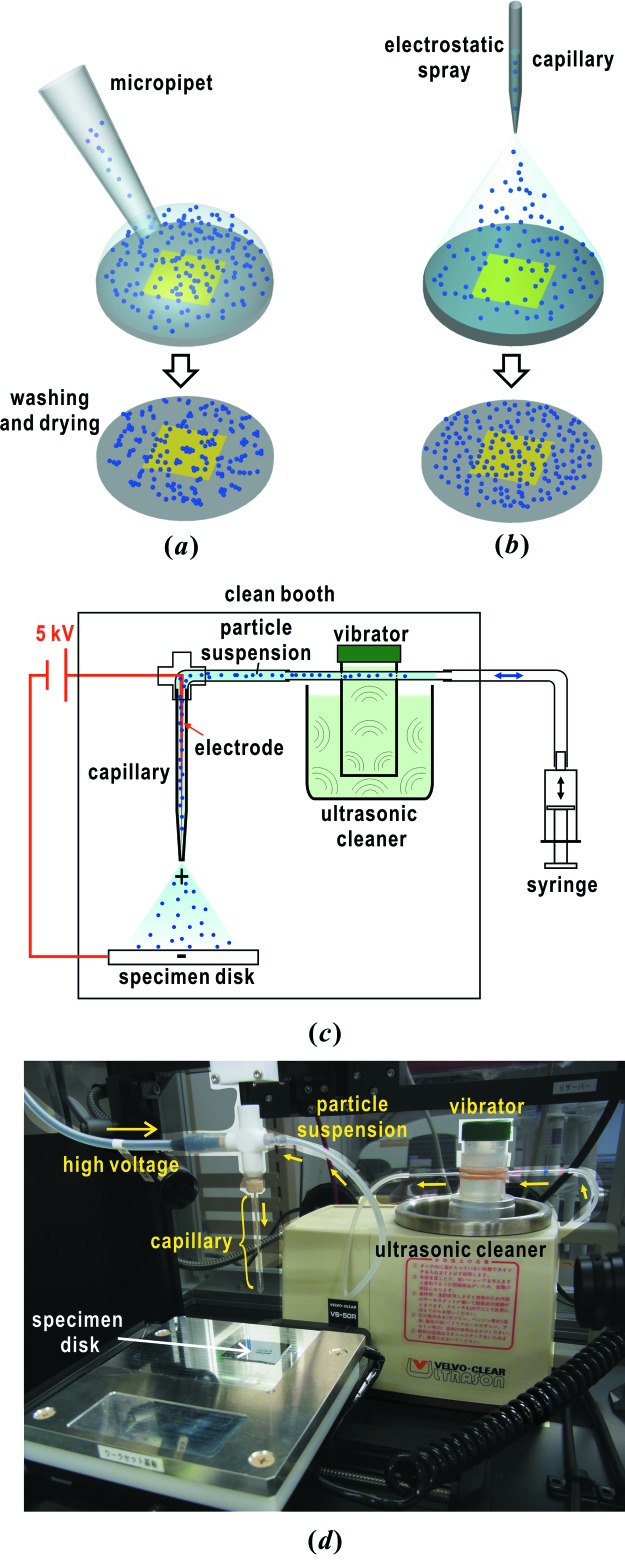
Schematic illustrations on the preparation of specimen disks carrying copper-oxide particles (*a*) and gold-colloidal particles (*b*). Schematic illustration (*c*) and a photograph (*d*) of our electrospray system used for uniformly dispersing gold-colloidal particles and PLL solutions. A thin electrode is inserted into a capillary tube with an inner diameter of 24 or 60 µm filled with 8 µL of concentrated suspension of gold-colloidal particles. The tip of the capillary nozzle is set about 10 mm apart from the specimen disk, and then the device is operated by applying a voltage of 5 kV between the electrode and the base plate. The specimen suspension in the capillary tube is evacuated to the vibrator to be dispersed by the ultrasonic cleaner for every 10 min operation to prevent heavy clogging by gravity sedimentation at the tip of the capillary.

In the cryogenic XFEL-CXDI experiments in this study, prior to diffraction data collection of biological specimens, we determine the camera parameters by analyzing diffraction patterns from single copper-oxide particles of cuboid shape (Kuo *et al.*, 2007 ▸) through the protocol we proposed earlier (Sekiguchi *et al.*, 2014*a* ▸). A 2 µL droplet of a concentrated suspension of copper-oxide particles is placed on the membranes using a micropipet (Fig. 3*a* ▸). After adsorption of the copper-oxide particles to the membranes, the suspension is washed out. Finally, the membranes are dried under atmosphere. We select specimen disks with a larger number of isolated particles through surveying the membrane surfaces using a scanning electron microscope.

In addition, we use gold-colloidal particles (British Biocell International Solutions, UK) with diameters of ∼50 nm to measure the spatial coherence of focused X-ray pulses through speckle visibility spectroscopy (Gutt *et al.*, 2012 ▸; Lehmkühler *et al.*, 2014 ▸). Details of the measurements and analytical methods for spatial coherence of XFEL pulses will be reported elsewhere.

We use the electrospray method (Oliver, 2004 ▸) to uniformly disperse the gold-colloidal particles on membranes without aggregates (Fig. 3*b* ▸). In this method, a charged droplet of suspension is electrically driven from a thin capillary tube. Then, the charged droplet is divided into many small mist particles by electrostatic repulsion. Finally, the gold-colloidal particles are directly fixed on the membranes through evaporation and electric breakup of water from the mist particles.

The gold-colloidal particles of diameter 50 nm are dispersed on membranes using a PDS-D01 electrospray device (Hamamatsu Nano Technology, Japan) (Figs. 3*b*–3*d* ▸). To avoid clogging of the gold-colloidal particles at the tip of the capillary tube of inner diameter 24 µm, the device is equipped with another custom-made device combining an ultrasonic cleaner and a syringe tube (Figs. 3*c*–3*d*) ▸.

### 2.3. Membranes with regularly arrayed PLL spots   

**Figure 4:**
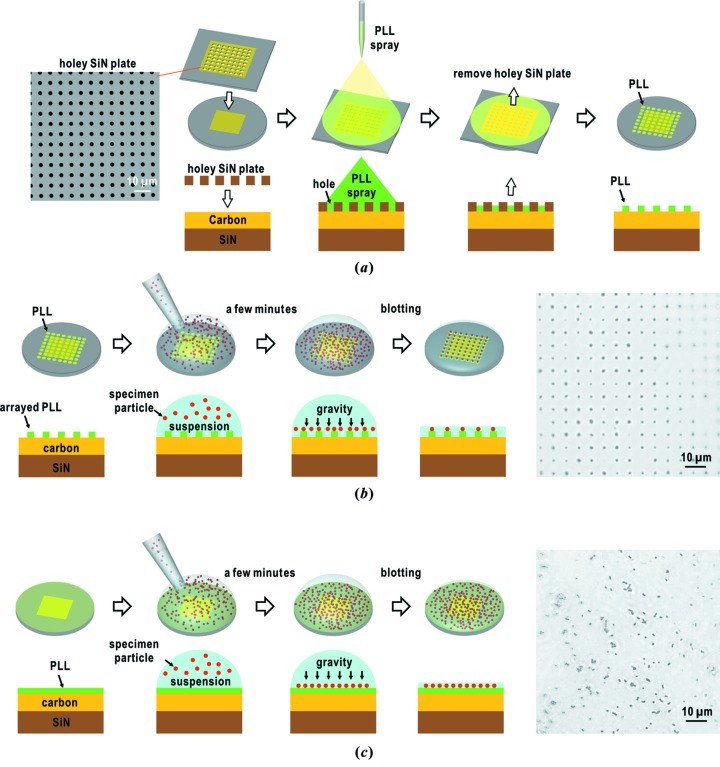
(*a*) Schematic illustration of the procedure to print regularly arrayed PLL spots onto carbon-coated SiN membranes by electrospraying the PLL solution with a commercially available holey SiN plate. The scanning electron micrograph of the holey SiN plate is displayed on the left (Norcada, Canada). Schematic illustrations of dispersing biological specimens, such as cyanobacteria cells on membranes with regularly printed PLL spots (*b*) and with a uniform PLL layer (*c*). The photographs on the right were taken after blotting by using the optical microscope IX71 in the phase-contrast mode.

If specified areas on the carbon or carbon-coated SiN membranes are decorated with PLL, biological specimens are adsorbed only onto the PLL-decorated areas. To prepare such a membrane, we electrospray a 0.1 mg ml^−1^ PLL solution through a capillary tube of inner diameter 60 µm onto the carbon or carbon-coated SiN membranes masked by a holey SiN plate of thickness 200 nm (Norcada, Canada) (Fig. 4*a* ▸). The plate has regularly arrayed 3 µm-diameter pinholes with a period of 6.4 µm. On these membranes with regularly decorated PLL spots, biological particles are adsorbed only onto the spots after blotting off the suspension (Fig. 4*b* ▸). In contrast, when a droplet of the suspension of biological particles is applied to the membranes decorated uniformly with PLL, the particles are adsorbed randomly onto the membranes (Fig. 4*c* ▸).

### 2.4. Preparation of frozen-hydrated biological specimens   

**Figure 5:**
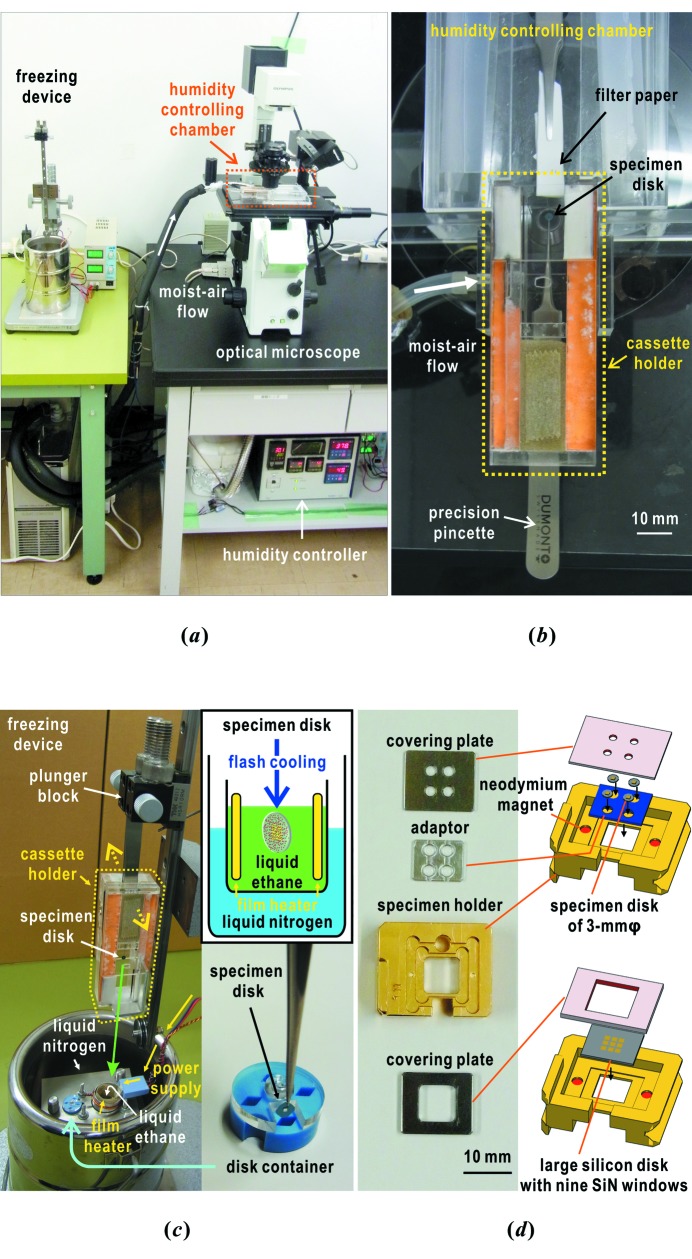
(*a*) Photograph of a set of devices used for preparing frozen-hydrated biological specimens, and a custom-made freezing device. (*b*) Magnified view of the humidity controlling chamber. A cassette holder is filled with moist air from the HUM-1, and is removable from the main body of the chamber. (*c*) Photograph of the flash-freezing device just before plunging the specimen disk into the liquid-ethane bath. Liquid ethane is produced by blowing ethane gas onto the wall of an aluminium cap cooled by liquid nitrogen. The temperature of liquid ethane is kept above the melting point by using a film heater of 7 Ω (Sakaguchi Dennetsu, Japan) connected to a power supply of 24 V as schematically illustrated in the inset on the upper right. The inset on the lower right is a photograph of a disk container to store flash-frozen specimen disks in liquid nitrogen (Yasuda Shoten, Japan). (*d*) Specimen holder and plates used to bring specimen disks from the liquid-nitrogen bath to the vacuum chambers of diffraction apparatus. Plates shown in the left photograph are used as illustrated on the right. Two neodymium magnets of 1.5 mm diameter and 3 mm height are buried in the specimen holder. An aluminium adaptor for carrying four specimen disks of diameter 3 mm is fixed to the specimen holder by a covering plate with four holes. The other covering plate is used to fix a silicon specimen disk with nine SiN windows to the specimen holder.

Fig. 5(*a*) ▸ shows a set of devices used for preparing frozen-hydrated biological specimens. A humidity-controlling chamber is mounted on the specimen stage of an IX71 optical microscope (Olympus, Japan) (Takayama & Nakasako, 2012 ▸). A HUM-1 humidity-controller (Rigaku, Japan) supplies moist air to the chamber. A custom-made freezing device is used to flash-cool specimens in liquid ethane. Through centrifugation, the concentrations of specimen particles in suspension are adjusted to yield finally a number density of approximately eight particles per 10 µm × 10 µm area of the membrane.

A specimen disk caught at the tip of a precision pincette is set in a removable cassette holder of the humidity-controlling chamber (Fig. 5*b* ▸). The interior of the cassette holder is filled with moist air from the HUM-1 controller. A 1–2 µL droplet of specimen suspension is applied to the membrane of the disk under a relative humidity (RH) higher than 90%. After a few minutes, sufficient for adsorption of specimen particles onto the membrane, an excess amount of suspension is blotted off using the tip of wick paper (MiTeGen, USA) or filter paper (Whatman, USA) under inspection by optical microscope.

When the number density of specimen particles and the amount of remaining buffer solution are within a desired range, the cassette holder is removed from the chamber. Then, we set the holder onto the freezing device, which is composed of a plunger block and a liquid-ethane bath (Fig. 5*c* ▸). The RH inside the holder is kept higher than 90%. Immediately after the pincette is fixed to the plunger block, the specimen disk at the tip of the pincette is dropped into the liquid-ethane bath. The flash-frozen specimen disks are stored in liquid nitrogen using disk containers (Yasuda Shoten, Japan) until used in the cryogenic XFEL-CXDI experiments (Fig. 5*c* ▸). In the experiments, specimen disks are fixed to custom-made specimen holders with a pair of small neodymium magnets in order to be delivered from the liquid-nitrogen bath to the vacuum chamber of the diffraction apparatus (Fig. 5*d* ▸). Four specimen disks of diameter 3 mm are set on an adaptor, and then a covering plate with four windows fixes the adaptor to the specimen holder. The large silicon specimen disk with nine SiN windows (10 mm × 8 mm) is fixed to the specimen holder by a covering plate made of stainless steel attracted by the neodymium magnets.

In this study, we used a cyanobacterium as a representative biological specimen. Cyanobacteria of *Prochlorococcus* strain NIES-2087 were purchased from the National Institute for Environmental Studies, Japan. The size distribution of cyanobacteria in the PRO-99 culture medium (Moore *et al.*, 2007 ▸) was measured by means of the dynamic light scattering (DLS) method by using a Zetasizer Nano S (Malvern, UK). The cyanobacterial cells of approximately 800 nm size are smaller than the focused spot sizes of the XFEL pulses (approximately 1–2 µm) available at SACLA (Yumoto *et al.*, 2013 ▸; Nakasako *et al.*, 2013 ▸; Oroguchi *et al.*, 2015 ▸).

### 2.5. Cryogenic XFEL-CXDI experiments at SACLA   

Cryogenic XFEL-CXDI experiments were performed at BL3 of SACLA (Tono *et al.*, 2013 ▸) using the KOTOBUKI-1 diffraction apparatus (Fig. 1*b* ▸) (Nakasako *et al.*, 2013 ▸; Sekiguchi *et al.*, 2014*b* ▸) or the TAKASAGO-6 diffraction apparatus (Fig. 1*c* ▸) (Kobayashi *et al.*, 2016 ▸). KOTOBUKI-1 is a prototype diffraction apparatus developed for examining the feasibility of cryogenic techniques introduced for CXDI experiments. The TAKASAGO-6 apparatus has been developed for high-throughput experiments based on the techniques developed using KOTOBUKI-1.

Each set of apparatus is equipped with a cryogenic pot on its goniometer inside the vacuum chamber. The temperature of the pot is kept at 66 K by the evaporation cooling effect (Nakasako *et al.*, 2013 ▸; Kobayashi *et al.*, 2016 ▸). Specimen disks fixed to specimen holders are transferred from a liquid-nitrogen bath to the cryogenic pot using a carrier and an automated loading device without frosting and warming (Nakasako *et al.*, 2013 ▸; Oroguchi *et al.*, 2015 ▸; Kobayashi *et al.*, 2016 ▸).

X-rays of wavelength 0.225 nm (X-ray photon energy of 5.5 keV) are focused to give intensities of 10^10^–10^11^ photons µm^−2^ pulse^−1^ by focusing mirror optics (Yumoto *et al.*, 2013 ▸). Each diffraction apparatus is placed so that the specimen position is within the beam waist of the focused X-ray pulses (Oroguchi *et al.*, 2015 ▸; Kobayashi *et al.*, 2016 ▸). Diffraction patterns are recorded by two multiport CCD (MPCCD) detectors (Kameshima *et al.*, 2014 ▸) in a tandem arrangement (Fig. 1 ▸). A MPCCD Octal detector placed approximately 1.6 m downstream from the specimen position is composed of eight CCD sensor panels and records diffraction patterns in the resolution range approximately 7–210 nm. The central aperture of the MPCCD Octal detector can be changed from a 3 mm square to a 9 mm square. A MPCCD Dual detector with two sensor panels is placed 3.2 m downstream of the specimen position with a beam stop of 2 mm × 2 mm. The Dual detector records small-angle diffraction patterns in the resolution range approximately 80–500 nm by attenuating the diffraction intensity using a set of aluminium films of thickness 15–100 µm. The small-angle resolution of approximately 500 nm can be achieved through eliminating the parasitic scattering from upstream optics by adjusting the positions of silicon-blade slits (Oroguchi *et al.*, 2015 ▸).

Diffraction patterns are collected through raster-scanning the specimen disks in steps of 25 µm in order to provide fresh specimen particles in the irradiation area. By using the high-precision goniometer of the prototype KOTOBUKI-1 apparatus, specimen disks of 3 mm diameter (Figs. 2*a* ▸ and 5*d* ▸) are raster-scanned synchronously with the motion of a pulse selector upstream of the beamline at a repetition rate of either 1 Hz or 2 Hz. The high-speed translation stage of the TAKASAGO-6 apparatus enables us to raster-scan the specimen disks for every XFEL pulse provided at a repetition rate of 30 Hz. We can utilize both large disks with nine SiN windows and disks of 3 mm diameter (Figs. 2*a* ▸ and 5*d* ▸) when using TAKASAGO-6.

### 2.6. Data processing and structure analysis   

A large number of diffraction patterns collected in each raster scan are processed by the *G-SITENNO* suite (Sekiguchi *et al.*, 2014*a* ▸,*b* ▸) on the SACLA HPC supercomputer system (Joti *et al.*, 2015 ▸). The *G-SITENNO* suite automatically subtracts dark current of the detectors from diffraction patterns, selects diffraction patterns with good signal-to-noise ratios at a desired resolution, and merges diffraction patterns recorded by the two MPCCD detectors. In addition, the suite preliminary provides projection electron density maps of the specimen particles by applying the phase-retrieval algorithm to selected diffraction patterns (Oroguchi & Nakasako, 2013 ▸) on the Mini-K supercomputer system (Joti *et al.*, 2015 ▸). After the data processing, *G-SITENNO* reports statistics of the diffraction data sets, and provides a compiled summary of diffraction patterns and retrieved projection images.

Further structure analyses are conducted for selected high-quality diffraction patterns of single particles which show good signal-to-noise ratios and maximum resolutions. The analyses are carried out using our structure analysis scheme *ASURA* (Sekiguchi *et al.*, 2016 ▸) on the Mini-K supercomputer system. In the first step, we determine the most probable overall shape of a particle, *i.e.* the support, by using the hybrid-input–output algorithm (Fienup, 1982 ▸) and the shrink-wrap algorithm (Marchesini *et al.*, 2003 ▸) according to the procedure reported previously (Kodama & Nakasako, 2011 ▸; Oroguchi & Nakasako, 2013 ▸). In the next step, the most accurate electron density distribution inside the determined support is estimated by applying the oversampling-smoothness algorithm (Rodriguez *et al.*, 2013 ▸). In each step we objectively select the most probable electron density maps by applying multivariate analysis to approximately 1000 electron density maps retrieved starting from different initial densities. Finally, we calculate a map as the average of the most probable class in the multivariate analysis. The effective resolution of the projection map is estimated by the phase-retrieval transfer function (PRTF) (Chapman *et al.*, 2006*b* ▸; Oroguchi *et al.*, 2015 ▸; Sekiguchi *et al.*, 2016 ▸).

When diffraction patterns lose a lot of speckles around the zero-diffraction angle because of saturation of the CCD detectors, we apply the dark-field phase-retrieval algorithm under the constraint of central symmetry in diffraction patterns (Kobayashi *et al.*, 2014 ▸).

## 3. Results   

### 3.1. Handling of specimen disks   

The three types of membranes used in this study are capable of withstanding flash-cooling. Carbon and epoxy membranes on molybdenum specimen disks of diameter 3 mm (Fig. 2*a* ▸) are mainly used for preliminary examination of the specimens. In contrast, the large silicon disks with nine windows (Fig. 2*a* ▸) are suitable for collecting a large number of diffraction patterns of specimens, the preparation conditions of which are well established.

In addition, the simplified procedure utilizing specimen holders with the pair of magnets, adaptor and covering plate is advantageous for avoiding problems such as the destruction of membranes by the tip of the pincette (Fig. 5*d* ▸). Mounting four small specimen disks on the specimen holder by use of the adaptor takes approximately 5 min in liquid nitrogen. The large SiN specimen disk is fixed to the specimen holder within 15 s.

### 3.2. Diffraction patterns from membranes   

**Figure 6:**
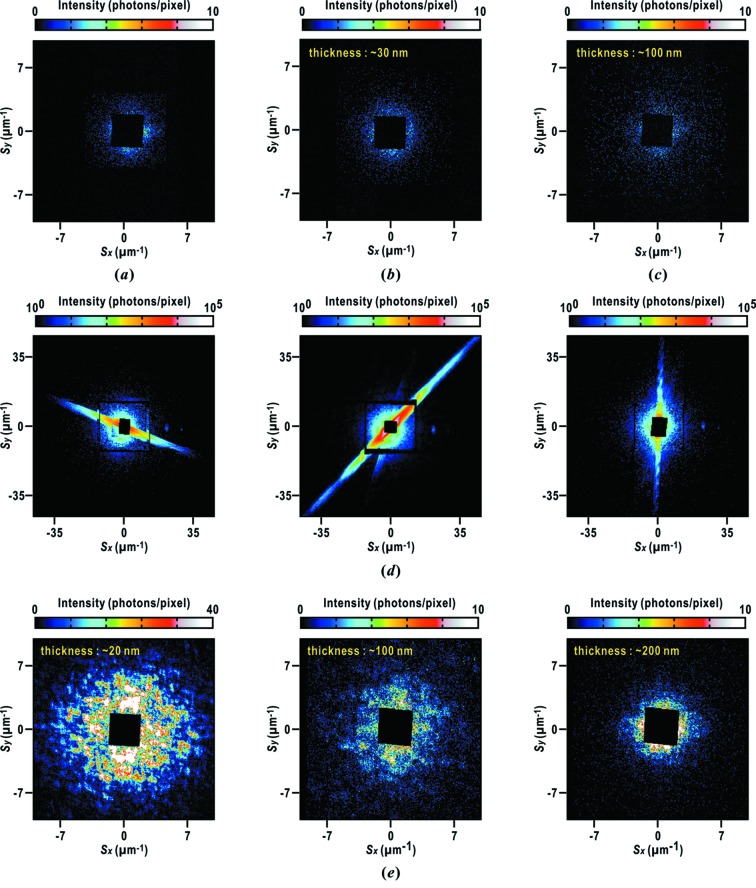
(*a*) Pattern of parasitic scattering around the beam stop coming from optics upstream. Diffraction patterns from a carbon membrane of thickness ∼30 nm (*b*) and a SiN membrane of thickness ∼100 nm (*c*). *S* is the scattering vector and defined as 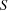 = 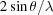, where 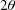 is the diffraction angle and λ is the X-ray wavelength. (*d*) Typical patterns from cracked membranes. Each pattern is approximated as Fraunhofer diffraction of a narrow slit. Evaluated from the speckle patterns, the width of each slit is estimated to be approximately 90 nm (left panel), 100 nm (center) and 300 nm (right). (*e*) Diffraction patterns from epoxy membranes with thickness of ∼20 nm (left panel), 100 nm (center) and 200 nm (right).

Diffraction patterns from the three types of membranes are compared with parasitic scattering from the upstream optics after tuning the positions of a pair of slits and the beam stop (Fig. 6*a* ▸). Diffraction intensities from carbon and SiN membranes are approximately less than five X-ray photons per pixel in small-angle regions around 3 µm^−1^, and almost invisible beyond 5 µm^−1^ (Figs. 6*b* and 6*c* ▸). Some detector pixels in the small-angle region close to the beam stop received 20 photons per pixel. However, the diffraction intensity is negligibly small in comparison with that from biological specimens exceeding 10^5^ X-ray photons per pixel. When recording diffraction patterns of the biological specimens, an aluminium attenuator placed in front of the MPCCD Dual detector completely absorbs the weak diffraction from the membranes as well as parasitic scattering. Carbon and SiN membranes occasionally crack over exposed windows by Coulomb explosion of particles on the membranes. These narrow and long cracks give strong and anisotropic streak patterns (Fig. 6*d* ▸). The tendency of cracking is likely prominent when the number density of particles is high.

Diffraction patterns from epoxy membranes vary depending on their thickness (Fig. 6*e* ▸). Epoxy membranes as thin as 20 nm give speckle peaks in the small-angle region up to a resolution of 8 µm^−1^. The speckle peaks suggest the presence of fluctuations in electron densities with approximate sizes of 200 nm, probably originating from planar micro domains in the epoxy polymers. On the other hand, speckle patterns become weak in membranes of thickness 100 nm. When the thickness of the membranes reaches 200 nm, speckle patterns disappear, indicating that the electron density projected along the incident X-rays becomes uniform by the increase in the thickness of the films. Therefore, we pay attention to controlling the thickness of epoxy membranes by adjusting the amount of reagents. In contrast to carbon and SiN membranes, epoxy membranes rarely crack even when particles are densely distributed in the irradiation area.

### 3.3. Standard specimens   

**Figure 7:**
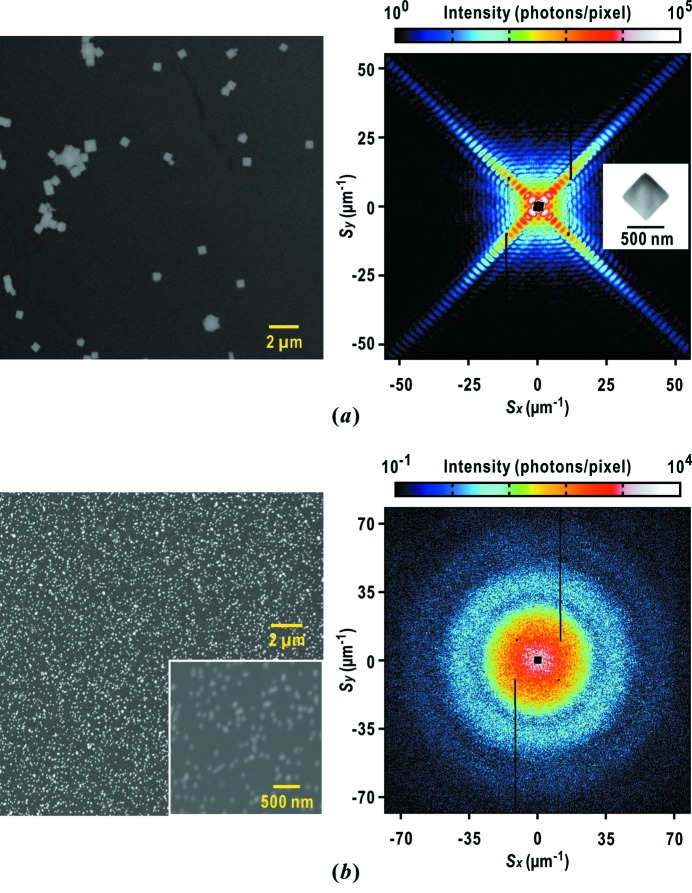
Preparation of standard specimens of metal particles. (*a*) SEM image of dispersed copper-oxide particles (left panel), and a diffraction pattern from a single copper-oxide particle of approximate size 500 nm. The electron density map projected along the direction of the incident X-ray pulse is retrieved as shown in the inset. We applied the *ASURA* protocol to the diffraction pattern up to a resolution of 25 nm at edge (Table 1 ▸). (*b*) SEM image and diffraction pattern of gold-colloidal particles of diameter 50 nm densely dispersed on a thin membrane.

It is difficult to apply the electrospray method to the suspension of copper-oxide particles, because the stabilization buffer contains a high concentration of reagents (33 m*M* sodium dodecyl sulfate, 200 m*M* sodium ascorbate and 1 *M* sodium hydroxide). When the suspension of copper-oxide particles is electrosprayed, powders of the three reagents appear on the membranes due to the evaporation and electric breakup of water from the sprayed mist of the suspension. Thus, we abandoned the application of the electrospray method for the suspension of copper-oxide particles. However, even from specimens containing aggregates of copper-oxide particles as identified in the scanning electron microscopy (SEM) images (Fig. 7*a* ▸), diffraction patterns from single copper-oxide particles (Fig. 7*a* ▸) are stochastically obtained at a rate of 11% against the total number of hits of XFEL pulses on single or clusters of particles during a raster scan. This efficiency is sufficient to determine the camera parameters.

For measuring the spatial coherence of the focused X-ray beam through visibility spectroscopy, specimen disks with uniformly and densely dispersed small gold-colloidal particles are necessary. The gold-colloidal particles in distilled water are uniformly dispersed without aggregates by using the electrospray device (Fig. 7*b* ▸). The diffraction patterns are composed of concentric rings, which originate from the overall shape of the gold-colloidal particles. The small speckle peaks originate from the interference of X-rays diffracted by particles in the irradiation area.

It takes 20 min until the number density of sprayed particles reaches 40 particles µm^−2^. In addition, the custom-made device is useful for preventing clogging caused by gravity sedimentation of the gold-colloidal particles (Figs. 3*c* and 3*d* ▸).

### 3.4. Diffraction patterns from frozen-hydrated cyanobacterial cells   

**Figure 8:**
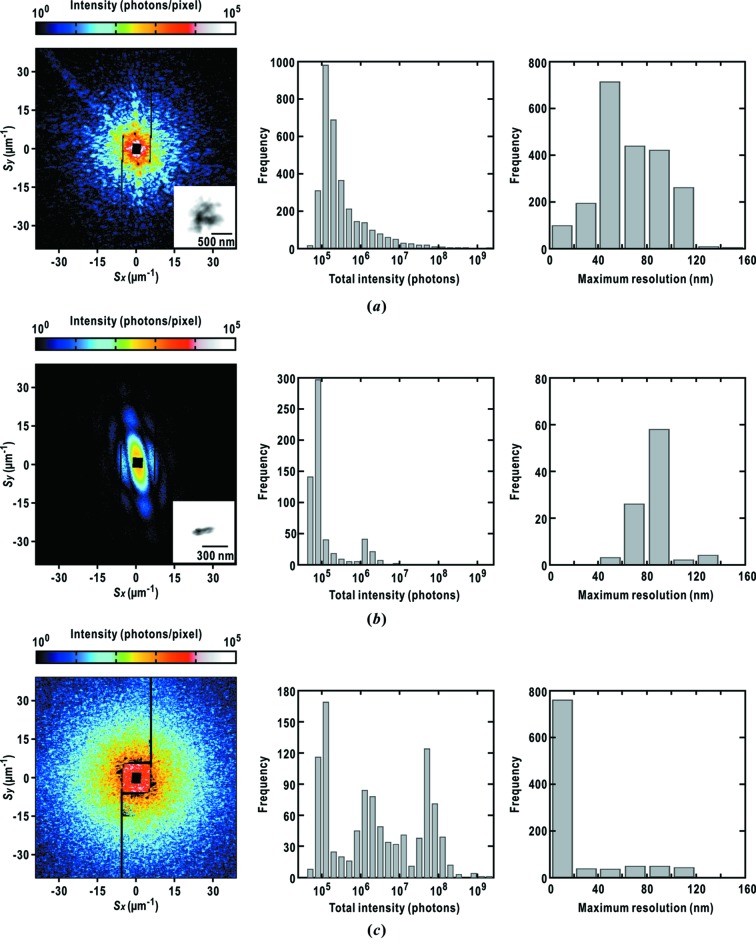
Diffraction patterns from flash-cooled cyanobacteria specimens with the frequency distribution regarding the total diffraction intensities and maximum resolutions. The total intensity of each diffraction pattern was calculated over the area displayed. The maximum resolution is defined as the highest-resolution shell including at least three detector pixels with more than four photons (Sekiguchi *et al.*, 2014*b* ▸). We did not analyze diffraction patterns with less than three detector pixels, which count more than four photons. The data shown are those for cyanobacteria surrounded by an appropriate amount of buffer solution (*a*), semi-dried (*b*) and heavily wet (*c*). The electron density maps retrieved from the diffraction patterns in Figs. 8(*a*) and 8(*b*) ▸ are shown in the inset. We applied the *ASURA* protocol to these diffraction patterns up to a resolution of 50 nm at edge. The statistics of the retrieved electron density maps are listed in Table 1 ▸.

The efficiency of diffraction data collection relies on the number density and dispersity of specimen particles. In the case of frozen-hydrated cyanobacterial cells, the average number density of approximately 8 particles per 100 µm^2^ results in a hit rate of approximately 35% against the total number of incident X-ray pulses. On the other hand, the quality of diffraction patterns depends on the amount of suspension remaining on the membranes (Fig. 8 ▸).

Fig. 8(*a*) ▸ displays a representative diffraction pattern from single cyanobacterial cell specimens with a diminished amount of suspension. The probability of obtaining such diffraction patterns is a few percent against the total number of XFEL pulses used in the raster scans. These patterns are composed of speckle peaks with sizes of ∼1.3 µm^−1^. They are comparable with the reciprocal sizes of single cyanobacterial cells of ∼780 nm. Among the diffraction patterns obtained through raster scans, the total diffraction intensities in the area displayed in Fig. 8(*a*) ▸ (40 µm^−1^ at the edge) is distributed from 10^5^ to 10^8^ photons, and the most frequently observed maximum resolution is ∼50 nm.

The diffraction pattern from a semi-dried cyanobacterial cell has an ellipsoid shape with long and short axes of approximately 300 nm and 90 nm, respectively (Fig. 8*b* ▸). The size is less than half of that shown in Fig. 8(*a*) ▸. In addition, the shape and size are inconsistent with those observed for *Prochlorococcus* strain MIT9313 in EM (Grassucci *et al.*, 2007 ▸). The statistics for the diffraction patterns collected in raster scans are different from those of the specimens shown in Fig. 8(*a*) ▸. The total diffraction intensities are of the order of 10^4^ photons, and the most frequently observed maximum resolution is approximately 100 nm. These findings suggest that evaporation of water would cause shrinkage of the cells and decrease the total cross section for X-rays.

When an excess amount of specimen suspension remains on the membranes, diffraction patterns are composed of speckles with smaller sizes than one-third of the reciprocal sizes of cyanobacterial cells (Fig. 8*c* ▸). Due to the large cross section in the irradiation area, most diffraction patterns have total diffraction intensities that are 10–1000 times higher than those from single cyanobacterial cells with a thin layer of suspension in Fig. 8(*a*) ▸, and then the maximum resolution of diffraction patterns reaches 20 nm. Taking the sizes of the object expected from speckle sizes, cells would overlap along the direction of the incident X-rays. A non-uniform thickness of vitreous ice within the irradiation area would also contribute to the diffraction patterns.

### 3.5. Projection electron density maps of cyanobacterial cells   

**Figure 9:**
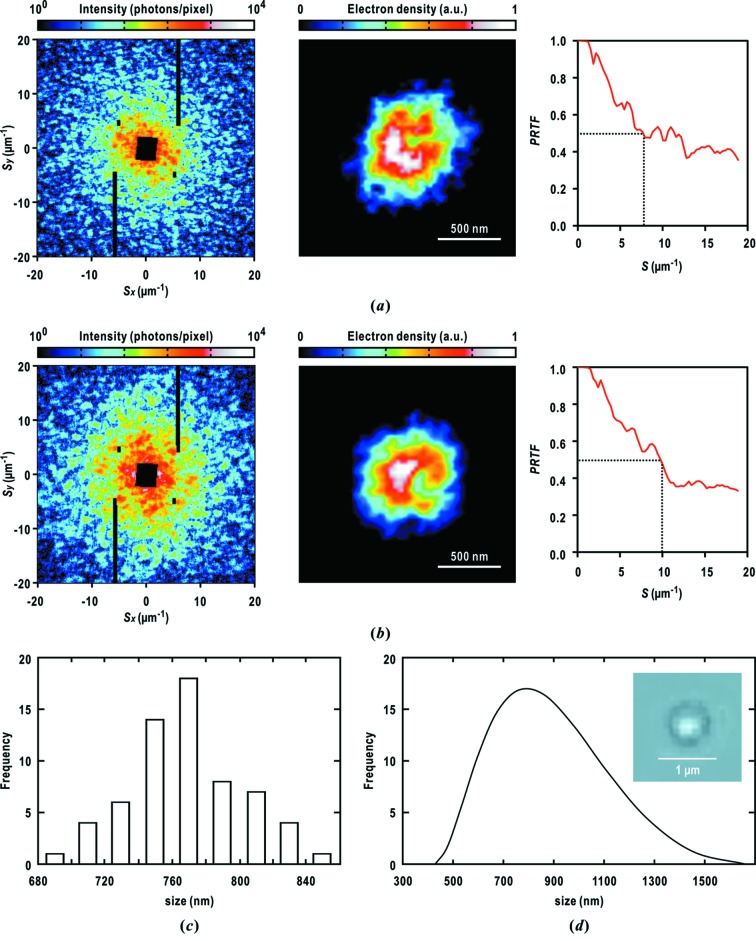
Two representative examples of diffraction patterns and the most probable electron density maps of frozen-hydrated single cyanobacterial cells. We applied the *ASURA* protocol to the diffraction patterns up to a resolution of 50 nm at edge. The statistics of the diffraction patterns and phase-retrieved projection electron density maps are summarized in Table 1 ▸. The effective resolutions of the density maps are estimated by using the PRTF. Size distribution of cyanobacterial cells for 63 retrieved projection electron density maps (*c*), and that measured by DLS (*d*). The inset shows an image of a cyanobacterial cell taken using an IX71 optical microscope.

Here we show diffraction patterns of flash-frozen cyanobacterial cells on a large SiN membrane. Through raster scans of 14512 points, we selected 63 diffraction patterns, such as those shown in Figs. 9(*a*) and 9(*b*) ▸, as being suitable for structure analyses with respect to the speckle size, diffraction intensities and maximum resolutions. The evaluated centro-symmetry scores of the diffraction patterns are better than 0.8 (Table 1 ▸), and then the speckle peaks of sizes 1.2–1.4 µm^−1^ are visible beyond resolutions of 20 µm^−1^. The isotropic distribution of speckle patterns suggests globular shapes of the frozen-hydrated cyanobacterial cells.

Through the *ASURA* protocol, the 63 projection electron density maps were retrieved at effective resolutions of better than 150 nm (Figs. 8*a* ▸, 9*a*, 9*b* ▸ and Table 1 ▸). Fig. 9(*c*) ▸ shows the distribution of cellular size measured for electron density regions higher than 0.25. The distribution has a peak centered at 775 nm with a standard deviation of 38 nm (Fig. 9*c* ▸). The peak value agrees with the cell size estimated by the DLS measurement prior to the specimen preparation (780 ± 230 nm). In addition, both results are consistent with the cell sizes observed by optical microscopy (Fig. 9*d* ▸). These findings indicate that the sizes of flash-frozen cyanobacterial cells would remain unchanged from those at ambient temperature.

In the phase-retrieved maps of cyanobacterial cells (Figs. 9*a* and 9*b* ▸), the electron densities inside the cells vary in the range 0.4–1.0, while those in the border regions of overall shapes are in the range 0.3–0.4. In particular, high-electron-density C-shaped regions with approximate sizes of 500 nm are sometimes observed. The C-shaped regions can be assigned as the stack of thylakoid membranes with reference to the tomography study of a frozen-hydrated cyanobacterial cell using cryogenic transmission EM (Ting *et al.*, 2007 ▸).

### 3.6. Diffraction data collection for specimen disks with regularly arrayed specimen particles   

We compared the efficiency of diffraction data collection between specimen disks with regularly arrayed particles (Fig. 4*b* ▸) and those with randomly dispersed specimen particles (Fig. 4*c* ▸). For randomly dispersed specimens with a number density of approximately 8 particles per 100 µm^2^, 1.2% of the total incident XFEL pulses yielded diffraction patterns suitable for structure analyses such as shown in Figs. 8(*a*) ▸, 9(*a*) and 9(*b*) ▸. In contrast, specimen disks with regularly arrayed particles recorded a hit rate of 2.4%. This difference is significant enough to increase the number of diffraction patterns suitable for structure analyses when millions of XFEL pulses are available in a beam time.

We rarely observed diffraction patterns from heavily wet specimens such as those shown in Fig. 8(*c*) ▸ when specimen disks with regularly arrayed PLL spots were used, probably because the surfaces of those membranes, except for the PLL spots, are hydrophobic to prevent them from wetting. In addition, the hydrophobic surfaces help us to easily remove the excess amount of suspension by blotting before flash-cooling. Therefore, specimen disks with regularly arrayed particles would be advantageous for collecting high-quality diffraction patterns from well characterized specimen particles. In contrast, because it is easy to prepare membranes uniformly decorated by PLL, specimen disks with randomly dispersed specimen particles are suitable for preliminary trials of collecting a large number of diffraction patterns.

### 3.7. Diffraction from ice crystals of sub-micrometer size   

**Figure 10:**
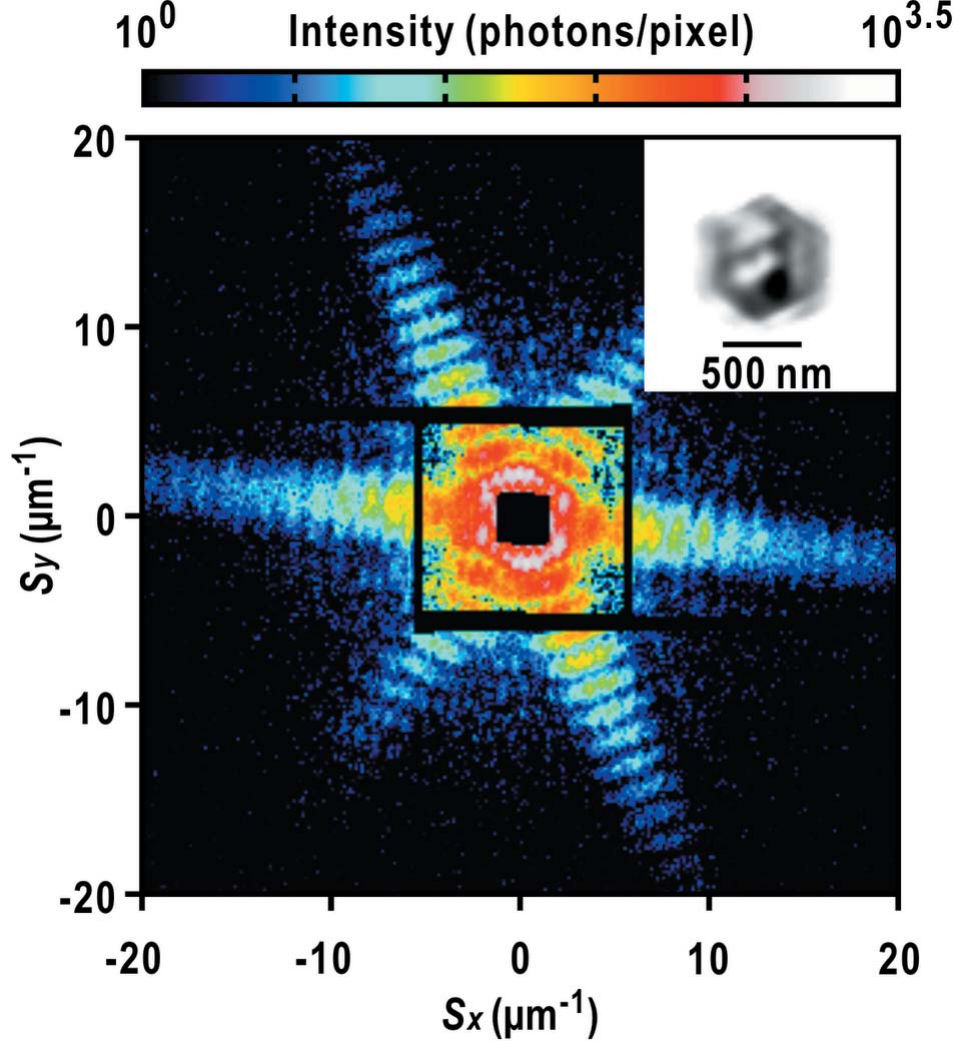
Diffraction pattern of a hexagonal ice crystal with sub-micrometer dimensions. The inset shows a retrieved electron density map projected along the direction of the incident X-rays.

If frosting occurs during the handling of cooled specimens (Fig. 5*c* ▸) or the temperature of specimens rises beyond the glass transition temperature of the specimen buffer (probably 150–180 K), a lot of hexagonal or cubic ice crystals appear causing intense streak patterns as shown in Fig. 10 ▸. We observed streak patterns quite rarely at a probability of less than 0.003% during our experiments in 2014 and 2015. Therefore, the procedures described here enable us to prepare frozen-hydrated biological specimens almost free from ice crystals.

## 4. Discussion   

We have developed membranes, devices and procedures to prepare frozen-hydrated biological specimens and standard metal specimens for cryogenic XFEL-CXDI experiments. In addition, the quality of the prepared specimens was examined through diffraction experiments. Based on the present results, here we discuss future developments in specimen preparation, and the characteristics of frozen-hydrated biological specimens in CXDI experiments.

### 4.1. Future development in specimen preparation   

As demonstrated in Fig. 8 ▸, an appropriate amount of buffer solution remaining around specimen particles is one of the important factors for collecting diffraction patterns suitable for structure analyses. Control of the amount of suspension through blotting depends on the contact modes of wick papers or filter papers with droplets of suspension, such as shape, period and direction. We currently control the amount of remaining buffer solution through inspection with optical microscope. However, semi-dried and heavily wet specimens appear depending on personal habits and skills in blotting. For specimen preparation without failures regarding the amount of buffer solution remaining around the specimen particles (Fig. 8 ▸), any robotics are particularly necessary to automatically control the blotting procedure with wick papers of regularized shapes and subsequent flash-freezing in liquid ethane. In addition, on the specimen disks with the arrayed PLL spots, an excess amount of specimen suspension can be removed easily by blotting, because the membrane surfaces without PLL spots are hydrophobic.

In raster scanning randomly dispersed biological particles, the probability of encountering diffraction patterns suitable for structure analyses (Figs. 8*a* ▸, 9*a* and 9*b* ▸) is a few percent of all diffraction patterns. Therefore, an increase in the hit rate is necessary for diffraction data collection in a limited beam time. In this regard, successful data collection for specimen disks with regularly arrayed spots of PLL improves the efficiency of collecting diffraction patterns suitable for structure analyses.

The best method for stochastically collecting a large number of high-quality diffraction patterns is to increase events that X-ray pulses hit specimen particles in a limited beam time. In our cryogenic experiments, the most time-consuming procedure is the exchange of the specimen holders. In this regard, the large specimen disks with multiple windows are advantageous for reducing the times of specimen exchanges. For instance, by using the TAKASAGO-6 diffraction apparatus, we can collect approximately 12000 diffraction patterns from one large silicon disk with nine SiN windows within 15 min.

High-quality diffraction data are obtained when the centers of a particle and the focal spot of an XFEL pulse almost coincide. If specimen particles are ideally arrayed in the same pitch of raster scans and their positions are adjusted against focused XFEL pulses, almost all XFEL pulses would hit single particles. At SACLA, the positional fluctuation of the focused X-ray beam is as small as 0.4 µm at least for two days as we have reported (Oroguchi *et al.*, 2015 ▸). Owing to the positional stability, better coincidence between particles and incident XFEL pulses would be possible by reducing the spot size of PLL to less than 2 µm, and controlling more accurately the distances between the PLL spots.

Through the present study, we conclude that a mechanically controlled blotting technique for the large disks, which are decorated by precisely arrayed PLL spots of diameter less than 2 µm, would be advantageous for obtaining high-quality diffraction patterns of single specimen particles through the collection of millions of diffraction patterns.

### 4.2. Little thermal shrinkage and adiabatic expansion of cells during flash-cooling   

Water occupies 60–70% of the volume of cells. When cells are set in a vacuum at ambient temperature, it is difficult to deny the probability that cellular structures are destroyed through the evaporation and bubbling of water inside the cells. In contrast, specimens embedded in vitreous ice are kept in the frozen-hydrated state in a vacuum of approximately 10^−4^ Pa. The thermal shrinkage of the cytosols inside cells during flash-cooling would be as small as that of vitreous ice frozen from water. Cryogenic X-ray protein crystallography has shown that the linear thermal-expansion coefficient of a protein is 25 × 10^6^ K^−1^ (Nakasako, 1999 ▸). Therefore, the thermal shrinkage of cellular specimens is negligibly small. In this study, this point is confirmed by the fact that the sizes of frozen-hydrated cyanobacterial cells are consistent with those of cyanobacteria in solution measured by DLS at ambient temperature (Fig. 9*d* ▸).

In near-future experiments, frozen-hydrated biological cells or organelles harvested in a desired cell cycle would be subjected to two types of CXDI experiments. The first type is XFEL-CXDI experiments to obtain projection electron density maps of millions of cells using the TAKASAGO-6 apparatus equipped with a fast scan stage. The second type is cryogenic CXDI tomography experiments for single cells at synchrotron facilities (Rodriguez *et al.*, 2015 ▸) using the KOTOBUKI-1 apparatus equipped with a high-precision goniometer within the limitation of radiation damage (Howells *et al.*, 2009 ▸). Then, the two-dimensional electron density maps from the XFEL-CXDI experiments would be compared with the three-dimensional electron density maps from the CXDI tomography experiments. When biological cells have similar internal structures, this experimental scheme conducted for frozen-hydrated specimens would be helpful for visualizing the three-dimensional structures of cells. Therefore, cryogenic XFEL-CXDI experiments would contribute to visualize the structures inside cells and organelles dynamically varying in cell cycles.

### 4.3. Contrast in projection electron density maps   

In the projection electron density maps retrieved from diffraction patterns of specimen particles in vitreous ice (Fig. 9 ▸), the fine structures inside the cells are clearly visualized rather than their outer shapes. In contrast, the overall shapes are clear as seen in the projection electron density maps retrieved from diffraction patterns of the cyanobacteria under semi-dried conditions (Fig. 8*b* ▸) and in vacuum (van der Schot *et al.*, 2015 ▸).

**Figure 11:**
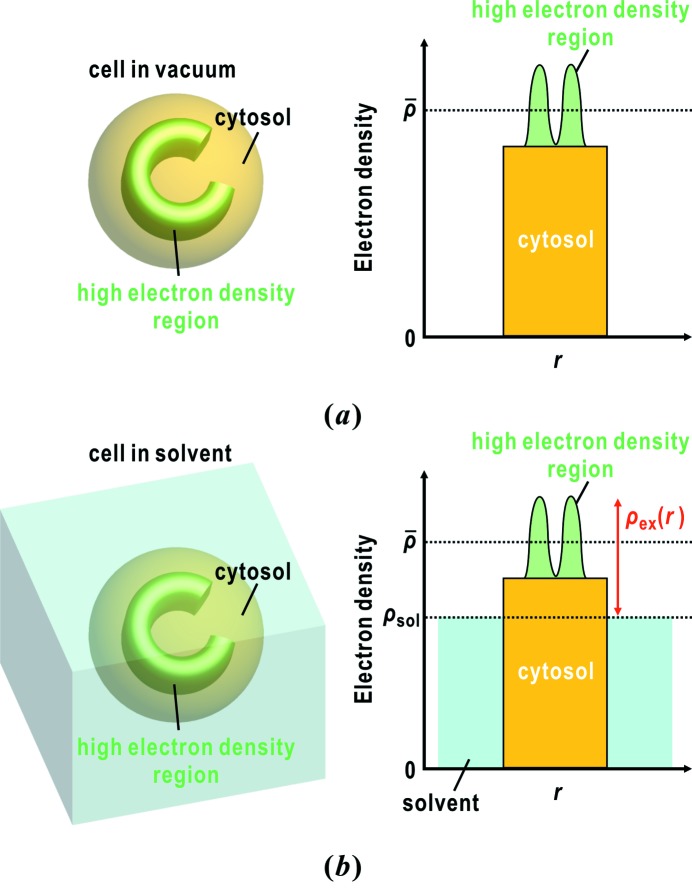
Schematic illustrations regarding the electron density contrast of biological cells in vacuum (*a*) and in solvent (*b*). The left panel shows a model system. The distribution of electron density in the model system is shown in the right panel.

The substantial differences between the retrieved electron density maps of cells in vacuum and in solvent can be explained by the contrast variation theory in small-angle X-ray scattering (Fig. 11 ▸) (Ibel & Stuhrmann, 1975 ▸). The excess electron density 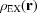 of a particle from the uniform electron density of solvent 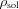 is expressed as
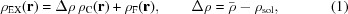
where 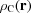 expresses the shape of the particle with average electron density 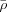. 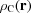 takes a value of 1 inside the particle and 0 outside the particle. 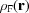 describes the spatial fluctuation of electron density from the average 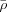. X-rays diffracted by solvent with 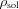 within the beam size of incident X-rays are hidden by a beam stop because of the reciprocity between the size and the scattering vector. Then, the diffraction intensity at a scattering vector 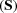, 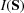, from 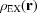 is expressed as
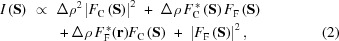
where 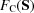 and 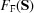 are the structure factors of 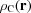 and 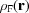, respectively. Because 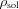 is almost zero for particles in vacuum, the diffraction intensity is dominated by the form factor from the overall shapes of specimen particles. In contrast, for specimen particles in vitreous ice or solvent, the diffraction intensity originating from the shape is reduced by the low contrast between 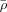 and 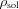. For instance, the 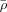 values of nucleic acids, proteins and water are 0.55, 0.42 and 0.33 electrons Å^−3^, respectively (Stuhrmann & Miller, 1978 ▸; Svergun *et al.*, 2013 ▸).

Therefore, biological particles in solvent or vitreous ice may be advantageous for extracting internal structures in the projection electron density maps. In the case of cyanobacteria, owing to the low contrast between the cells and the surrounding solvent, the structures inside cells, such as C-shaped high-electron-density regions, would appear clearly.

### 4.4. Application of the preparation method to biological specimens   

The specimen preparation procedure here has been applied to various biological specimens. Recently, we obtained projection electron density maps of yeast nuclei, magnetic bacteria and chloroplasts of algae (Oroguchi *et al.*, 2015 ▸; Takayama *et al.*, 2015 ▸), whose sizes are smaller than the focal spot size of 2 µm (Nakasako *et al.*, 2013 ▸; Kobayashi *et al.*, 2016 ▸). Specimens with small total scattering cross section give diffraction patterns with poor signal-to-noise ratio. Therefore, biological specimens of approximately 1 µm size are desirable for XFEL-CXDI experiments at SACLA, when taking into account both the incident X-ray intensity and the focal spot size.

The number density of biological particles dispersed on PLL-coated membranes depends on the electrostatic interactions between the particles and the amino group of PLL. Cyanobacterial cells show good affinity to PLL. However, the affinity depends on, for instance, the amount of membrane proteins and other biological molecules existing on cell surfaces. Therefore, reagents with different types of functional groups would be necessary in some cases.

## Figures and Tables

**Table 1:** Statistics of diffraction patterns and phase-retrieved electron density maps

	Fig. 7(*a*) ▸	Fig. 8(*a*) ▸	Fig. 8(*b*) ▸	Fig. 9(*a*) ▸	Fig. 9(*b*) ▸
Diffraction pattern					
*C*_sym_ †	0.72	0.85	0.46	0.86	0.81
Maximum resolution (nm)‡	11.1	20.0	50.1	13.5	17.6
Phase-retrieved electron density map					
Number of maps included in the most probable class in the analysis of the internal structures by the *ASURA* scheme	301	204	212	194	167
Oversampling ratio§	161.8	14.1	321.3	11.1	14.6
*R*_F_ ¶	0.18	0.31	0.34	0.33	0.32
Effective resolution (nm)††	18.2	136.9	18.2	126.4	99.6

† The Friedel symmetry of a diffraction pattern is evaluated using the following correlation: 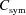 = 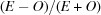, *E* = 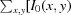 + 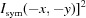, 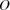 = 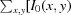 − 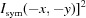, where 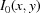 is the diffraction intensity in the region of interest with 100 × 100 pixels and 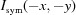 is the diffraction intensity of the Friedel mate. For a diffraction pattern with ideal Friedel symmetry, the 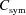 value becomes 1 (Sekiguchi *et al.*, 2014*a* ▸).

‡ The maximum resolution in a diffraction pattern is defined as the highest-resolution shell including at least three detector pixels with more than four photons (Sekiguchi *et al.*, 2014*b* ▸).

§ The oversampling ratio of the density model is defined as the ratio between the number of pixels in the determined support in the electron density maps and the number of pixels in the diffraction patterns.

¶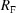 is defined as 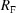 = 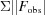 − 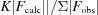, where 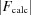 and 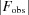 represent the structure amplitude calculated from the reconstructed electron density and observed in experiments, respectively. *K* is a scale factor between the reconstructed and observed structure amplitudes (Miao *et al.*, 2005 ▸). In protein crystallography, an 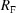 value smaller than 0.2 is desirable to confirm that the electron density maps display structures up to atomic resolution after crystallographic refinement. On the other hand, initial electron density maps suitable for building polypeptide models frequently show an 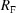 value larger than 0.4. In our studies, the phase-retrieval procedure is not applied to diffraction data up to atomic resolution. Therefore, we currently accept electron density maps of 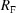 values 0.3–0.4 as successfully reconstructed maps. In fact, an 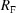 value of ∼0.3 is commonly used for a reasonable starting model for refinement as reported in X-ray crystallography (Murshudov *et al.*, 2011 ▸).

†† The effective resolution of the averaged electron density map is defined as the resolution where PRTF drops below 0.5 (Chapman *et al.*, 2006*b* ▸; Sekiguchi *et al.*, 2016 ▸). PRTF is calculated from electron density maps of the most probable class selected in the multivariate analysis.
